# The impact of hospital-specific guidelines on carbapenem use and patient outcomes in a setting for high endemicity with multidrug-resistant gram-negative bacilli

**DOI:** 10.1017/ash.2024.415

**Published:** 2024-09-25

**Authors:** Cemre Boşnak, Şeyda Betül Fındık, Muhammed Atay, Ward Fakhouri, Sada Babazade, Eda Karadoğan, Gökhan Metan, Ömrüm Uzun

**Affiliations:** 1 Department of Infectious Diseases and Clinical Microbiology, Hacettepe University, Ankara, Turkey; 2 Faculty of Medicine Graduate Program, Hacettepe University, Ankara, Turkey; 3 Faculty of Medicine Department of Public Health Division of Epidemiology, Hacettepe University, Ankara, Turkey

## Abstract

**Objective:**

This study aims to assess the impact of hospital-specific guidelines on the optimal utilization of carbapenems and to examine their effects on patient outcomes.

**Design:**

Quasi-experimental study.

**Setting:**

Tertiary care hospital in Turkey where infectious diseases (IDs) consultation and antibiotic approval are mandatory for carbapenem use.

**Participants:**

All inpatients ≥18 years of age who received a carbapenem for at least 24 hours during the study periods were enrolled.

**Intervention:**

Hospital-specific treatment guidelines were introduced in April 2019. The control group was the year 2018, when there were no guidelines (pre-GP). The year 2020 was analyzed as the intervention period (post-GP).

**Results:**

A total of 678 patients were analyzed, 326 in the pre-GP period and 352 in the post-GP period. Following guideline implementation, there was a significant increase in appropriate carbapenem use (49.1% in pre-GP vs 71.9% in post-GP, *P* < .001). The duration of carbapenem use decreased significantly (*P* = .019). However, there was no significant change in the incidence of new infection episodes within the subsequent 30 days (27.6% in pre-GP vs 28.3% in post-GP), or in the length of hospitalization [median (25%–75%) = 28 (16–46) in pre-GP, 28 (15–47.5) in post-GP, *P* = .678]. Mortality rates were similar at day 7 post-GP (1.7%) compared to pre-GP (0.03%) (*P* = .125).

**Conclusions:**

The implementation of guidelines increased the appropriate utilization of carbapenems, without resulting in extended hospital stays or recurrent episodes. Despite an increased number of patients admitted to the ICU during the latter period, infection-related mortality rates remained comparable.

## Introduction

Antimicrobial resistance has emerged as a serious public health threat. Carbapenems serve as an important choice in treating bacterial infections caused by multidrug-resistant (MDR) bacteria because of their wide spectrum of activity against bacteria with extended-spectrum beta-lactamase (ESBL) and Amp-C enzymes. In the 2021 Global Antimicrobial Resistance and Use Surveillance System report, carbapenems were included in the ’watch list’ of antibiotics and were recommended to be protected as the last line of defense due to the risk of resistance development.^1^

Treatment of infections caused by carbapenem-resistant pathogens is challenging due to the limited availability of effective antimicrobials, thus judicious carbapenem use is essential to prevent the emergence of carbapenem resistance. Several studies focusing on the use of carbapenems have often included audit and feedback within the context of the Antimicrobial Stewardship Programme.^2–7^ To the best of our knowledge, no study has evaluated the effect of a hospital-specific guideline alone on the appropriate use of carbapenems and its impact on patient outcomes.

In our hospital, infection disease consultation and approval of certain broad-spectrum antibiotics are the standard. To ensure standardization among consultants, hospital-specific guidelines were developed in 2019. In this study, we compared the pre-guideline period (pre-GP) with the post-guideline period (post-GP) in terms of appropriateness of carbapenem prescribing practices and their effect on patient management.

## Materials and methods

### Study design

The study was designed as a quasi-experimental study. Hospital-specific treatment guidelines were introduced in April 2019. The guidelines can be accessed in English via the link ’https://enfeksiyon.hacettepe.edu.tr/en’. Updates were communicated both verbally and in writing. Using guidelines was made mandatory for all infectious disease (ID) physicians. Patients who used carbapenems for any infection in 2018 (pre-GP) were included as the control group, and those in 2020 (post-GP) were the intervention group. In addition to the guideline, a future study will evaluate the effect of the feedback audit after 2020.

### Clinical setting

Hacettepe University Hospital has 1,040 ward beds, six intensive care units (ICUs) with a total of 143 beds.

An infection prevention and control plan for the management of patients colonized or infected with MDR organisms was already in practice at the time of the study. Throughout the study period, clinical specimens for microbiological evaluation were collected at the bedside. Blood samples were processed using the BACTEC 9240 blood culture system (Becton Dickinson, Cockeysville, MD, USA). Bacterial species isolated from all samples were identified using Matrix-Assisted Laser Desorption/Ionization Time-of-Flight Mass Spectrometry and conventional tests. Antibiotic susceptibility profiles of the isolates were determined by BD Phoenix™ automated susceptibility testing system (Becton Dickinson and Company BD, USA), Kirby-Bauer disk diffusion susceptibility test, or antimicrobial gradient test. Antibiotic susceptibility results were interpreted according to European Committee on Antimicrobial Susceptibility Testing recommendations.^8^ Fecal samples were tested using a rapid immunochromatographic detection of *Clostridiodes difficile* antigen (glutamate dehydrogenase antigen), and toxins A and B.

The use of third-generation cephalosporins, piperacillin-tazobactam, glycopeptides, tigecycline, parenteral fluoroquinolones, aminoglycosides, and carbapenems has been restricted to the approval of ID physicians since 2003. ID consultation and Infectious disease approval are available for 7 days/24 h. ID team regularly visits all patients who are on antibiotics with restricted use until clinical stabilization is achieved.

### Patient population

All inpatients ≥18 years of age who received a carbapenem for at least 24 hours during the study periods were enrolled. Patients receiving empiric therapy required at least one set of blood cultures taken before initiation of antibiotic therapy and samples from suspected sites of infection. Only the first episode of carbapenem treatment per patient was included. Exclusion criteria were: use of carbapenems based on culture results (pre-GP n = 38, post-GP n = 58), no culture tests before treatment (pre-GP n = 22, post-GP n = 43), death within 24 hours of treatment (pre-GP n = 7, post-GP n = 16), initiation of treatment in another center (pre-GP n = 3, post-GP n = 45), and recurrent episodes of carbapenem use (pre-GP n = 13, post-GP n = 35).

## Outcome

The primary outcome was the appropriateness of carbapenem treatment defined according to the presence of risk factors for MDR bacteria infection. Duration of carbapenem use, transfer to the ICU, length of hospital stay, and mortality were assessed as secondary outcome measures.

### Data collection

Data for this study were collected retrospectively for the pre-GP (Jan 1^st^, 2018 to Dec 31^st^, 2018), and post-GP (Jan 1^st^, 2020 to Dec 31^st^, 2020) from electronic records in the hospital information system. Hospital-specific treatment guidelines were introduced in April 2019.

Data including colonization by *Candida* spp., carbapenem-resistant *Enterobacterales* (CRE), *C. difficile* infection, and new infection episodes in 30 days were recorded. Infection density rates for nosocomial bacteremia per 10,000 patient days caused by ESBL-producing *E. coli* (ESBL-EC) and *K. pneumoniae* (ESBL-KP), carbapenem-resistant *K. pneumoniae* (CRKP), *Acinetobacter baumannii* (CRAB), and *Pseudomonas aeruginosa* (CRPA), methicillin-resistant Staphylococcus aureus (MRSA), and Vancomycin-resistant *Enterococcus faecium* (VRE) in 2018 and 2020 were extracted from Hospital Infection Control Committee reports. The consumption rates of carbapenems, piperacillin-tazobactam, ampicillin-sulbactam, 3^rd^ and 4^th^ generation cephalosporins, fluoroquinolones (ciprofloxacin, levofloxacin, and moxifloxacin), amikacin, tigecycline, polymyxins, and glycopeptides were measured in days of therapy (DOT) per 1,000 patient days.

### Definitions

Carbapenem use was considered as appropriate if at least one of the following conditions was met:

1. History of a fluoroquinolone, 3^rd^ or 4^th^ generation cephalosporin, piperacillin-tazobactam, tigecycline, or carbapenem use for ≥7 days in the last 1 month
2. Sepsis in the ICU after ≥72 hours of ICU admission
3. Ventilator-associated pneumonia after ≥ 96 hours of ICU admission
4. Infection or colonization by an MDR gram-negative bacteria (GNB) resistant to non-carbapenem antibiotics in the last 3 months
5. Neutropenic patient with fever under levofloxacin prophylaxis
6. Patient with septic shock with a history of unknown recent antibiotic use.

In both study periods, the dosage of carbapenems was adjusted according to the renal function of the patient after the first day doses. Institutional guidelines recommend a three-hour prolonged infusion after the first dose of meropenem for patients treated in the ICU.

The patient was considered to be eligible for de-escalation from carbapenem treatment if the causative pathogen had a susceptibility profile that favored de-escalation, the patient was clinically stable and afebrile for ≥24 hours. Escalation was defined as broadening the empirical carbapenem regimen based on antimicrobial susceptibility results or clinical response.

Successful outcome was defined as the resolution of symptoms and signs of infection and improvement of relevant laboratory parameters. Death was attributed to the infectious process if it occurred within 7 days of carbapenem treatment. Crude all-cause mortality was defined in the 30-day follow-up period.

The source of the infection was established through the implementation of diagnostic criteria outlined by the Centers for Disease Control and Prevention (CDC).^9,10^ Sepsis was defined according to the Sepsis-3 criteria.^11^

### Sample size

The sample size was calculated with the Open epi program using the sample size for cohort-rct when the case/control ratio was calculated as 1/1, and delta value of 0.24 (percent of unexposed with outcome 0.39, percent of exposed with outcome 0.63) was used resulting in a minimum required number of 95 intervention group and 95 control group was achieved with 90% power, and all patients were included in the study to avoid loss of data.

### Statistical analysis

Descriptive statistics were presented as n (%) for categorical variables and as median (25–75 percentile) for continuous variables, as the continuous variables did not exhibit a normal distribution. The distribution of continuous data was assessed using the Kolmogorov–Smirnov test and histogram. The χ^2^or Fisher’s exact test was utilized for the comparison of categorical variables. Mann–Whitney test was used to compare continous data in two groups. Binary logistic regression was used for multivariate analysis of the effect of guideline implementation on appropriate carbapenem use, while adjusting for confounders. Charlson Co-morbidity Index (CCI), Coronavirus diseases 2019 (COVID-19) history, ICU stay and blood culture growth were considered as confounders when examining the effect of guideline implementation on appropriate carbapenem use. The presence of effect modification was also examined in the multivariate analysis; no effect modifier was identified. Because there was no COVID-19 in the pre-guideline period, the effect-modifying role of COVID-19 could not be evaluated. Cox regression analysis was used for multivariate analysis of the effect of guideline implementation on mortality, while adjusting for confounders. COVID-19, CCI, ICU, blood culture growth, new infection episode within 30 days were evaluated as confounders in mortality analyses. The effect size was reported as a risk ratio, odds ratio or hazard ratio with 95% confidence intervals.

Nosocomial bloodstream infection density rates and antibiotic consumption rate per 100 days [95% confidence intervals (CI)] for each period were calculated by using OpenEpi (Open-Source Epidemiologic Statistics for Public Health) version 3.01 (https://www.OpenEpi.com). The Mid-P exact test was used to compare infection density rates and antibiotic consumption rates in pre-GP and post-GP.

A two-tailed type 1 error rate of 0.05 was considered for all analyses. The analyses were conducted using the Statistical Packages for the Social Sciences (IBM SPSS Corp, Armonk, New York, USA) version 29 software package.

### Ethical issues

This study was approved by the Hacettepe University Clinical Research Ethics Board.

## Results

A total of 326 patients were included in the pre-GP and 352 in the post-GP (Table 1). Rate of admission to ICU (*P* < .001), and CCI was higher (*P* = .011) in post-GP. Urinary tract infection was more common in the pre-GP (34.7% vs 22.7%, respectively *P* < .001), whereas pneumonia was the leading infection in the post-GP (27.3% in the pre-GP vs 40.3% in the post-GP, *P* < .001). A total of 74 out of 351 patients (21%) had a diagnosis of COVID-19 in the post-GP.

**Table 1.** Demographic characteristics of patients in the pre- and post-guideline periods

	Pre-GP (N = 326)	Post-GP (N = 352)	*P* value
Age, years, median (25%–75%)	64.0 (49–73)	64.0 (55–75)	.323
Women, N (%)	143 (43.9)	153 (43.5)	.917
Comorbidities, N (%)			
Diabetes mellitus	90 (27.6)	103 (29.3)	.633
Hypertension	117 (35.9)	149 (42.3)	.086
Cardiovascular disease	86 (26.4)	98 (27.8)	.669
Chronic renal disease	32 (9.8)	32 (9.1)	.747
Cirrhosis	5 (1.5)	4 (1.1)	.744
Rheumatological disease	11 (3.4)	11 (3.1)	.855
Neurological disease	57 (17.5)	53 (15.1)	.392
Chronic pulmonary disease	41 (12.6)	46 (13.1)	.848
Solid tumor	117 (35.9)	134 (38.1)	.557
Hematological malignancy	46 (14.1)	38 (10.8)	.191
Hematopoietic stem cell transplantation (autologous and allogeneic)	11 (3.4)	6 (1.7)	.165
Solid organ transplantation	15 (4.6)	3 (0.9)	.002
COVID-19	–	74 (21.0)	<.001
Other	44 (13.5)	63 (17.9)	.116
Charlson comorbidity index (CCI), median (Interquartile range, 25%–75%)	4 (2–6)	5 (3–8)	.011
Patient in the ICU when carbapenem was started	84 (25.8)	202 (57.4)	<.001
Initial infectious disease diagnosis, N (%)			
Urinary tract infection	113 (34.7)	80 (22.7)	<.001
Pneumonia	89 (27.3)	142 (40.3)	<.001
Intraabdominal infections	55 (16.9)	53 (15.1)	.519
Neutropenic fever	42 (12.9)	46 (13.1)	.943
CNS infection	14 (4.3)	3 (0.9)	.004
Skin and soft tissue infections	18 (5.5)	25 (7.1)	.399
Sepsis with unknown origin	13 (4.0)	70 (19.9)	<.001
Bone and joint infection	4 (1.2)	4 (1.1)	1.000
Catheter-related infection	5 (1.5)	7 (2.0)	.654

Note. Cardiovascular diseases: coronary artery disease, heart failure, and atrial fibrillation. Rheumatological diseases: rheumatoid arthritis, lupus, ankylosing spondylitis, scleroderma, psoriatic arthritis, polymyositis. Neurological diseases: Alzheimer’s disease, Parkinson’s disease, multiple sclerosis, amyotrophic lateral sclerosis (ALS), epilepsy, and cerebrovascular stroke. Chronic lung diseases: comorbid asthma, chronic obstructive pulmonary disease (COPD), interstitial lung disease, or bronchiectasis. Solid tumors: breast cancer, lung cancer, colon cancer, brain tumors, stomach cancer, liver cancer. Hematological malignancy: AML, ALL, myelodysplastic syndromes (MDS), CML, and CLL. SOT (solid organ transplantation): includes liver and kidney transplantation. Other diseases: benign prostatic hypertrophy, hypothyroidism, viral hepatitis, HIV, hemophilia, inflammatory bowel disease, psychiatric diseases, biliary tract diseases, dermatological diseases, and neurogenic bladder. Urinary system infections: urethritis, prostatitis, cystitis, and pyelonephritis. Intra-abdominal infections: appendicitis, peritonitis, intra-abdominal abscess, cholecystitis, cholangitis, infections following trauma to abdominal organs, and bowel perforations. CNS infections: meningitis, encephalitis, and cranial abscess. Bone and joint infections: septic arthritis and osteomyelitis. Pre-GP: pre-guideline period; post-GP: post-guideline period; COVID-19: coronavirus disease 2019.

Empirical carbapenem use was more frequent in the post-GP. The frequency of bacteremic patients and carbapenem-resistant GNB isolation from the clinical samples were comparable in both periods. The rate of appropriate use of carbapenems increased significantly in the post-GP overall (49.1% in pre-GP vs 71.9% in post GP, *P* < .001), and in the subgroup of bacteremic patients (56.4% in pre-GP vs 77.4% in post-GP, *P* = .021). In a sub-analysis of 286 patients admitted to the ICU with renal dysfunction, correct dosing on the first day and three-hour prolonged infusion increased (63.4% in pre-GP vs 27.4% in post-GP, *P* < .001) (Table 2). When CCI, COVID-19 history, ICU stay, and blood culture growth were controlled by multivariate analysis, appropriate carbapenem use increased in the post-GP period [OR (95% CI) = 1.8 (1.3–2.6), *P* < .001] (Table 3). De-escalation of carbapenem treatment was more frequent (12.4% in the pre-GP vs 22.7% in the post-GP, *P* = .002), and the median duration of carbapenem use was shorter in the post-GP (8 days in the post-GP vs 9 days in the pre-GP, *P* = .019).

**Table 2.** Comparison of carbapenem use and treatment outcomes in the pre- and post-guideline periods

	Pre-GP (N = 326)	Post-GP (N = 352)	*P* value
Type of carbapenem treatment, N (%)			
Empirical	237 (72.7)	316 (89.8)	<.001
Based on culture result	89 (27.3)	36 (10.2)	
Type of prescribed carbapenem, N (%)			
Meropenem	232 (71.2)	324 (92.0)	<.001
İmipenem	1 (0.3)	2 (0.6)	
Ertapenem	93 (28.5)	26 (7.4)	
Initial treatment regimen, N (%)			
Carbapenem alone	231 (70.9)	241 (68.5)	.218
Combination regimen	95 (29.1)	111 (31.5)	.049
Carbapenem + glycopeptide	58 (17.8)	76 (21.6)	
Carbapenem + colistin/amikacin	16 (4.9)	13 (3.7)	
Carbapenem + colistin/amikacin + glycopeptide	3 (0.9)	9 (2.6)	
Carbapenem + other	18 (5.5)	13 (3.7)	
Appropriateness of carbapenem use, N (%)			
Adequate initial dosage in renal dysfunction^a^ and administration with prolonged infusion	23 (27.4)	148 (63.4)	<.001
Correct indication for carbapenem	160 (49.1)	253 (71.9)	<.001
Positive blood culture, N (%)	55 (16.9)	53 (15.1)	.519
Appropriate carbapenem use in bacteremia^b^	31 (56.4)	41 (77.4)	.021
Successful treatment of the infectious episode, N (%)	281 (86.2)	318 (90.3)	.093
Duration of carbapenem treatment, days, median (25%–75%)	9 (6–12)	8 (5–11)	.019
Modification of initial carbapenem therapy			
Escalated, N (%)	122 (37.4)	63 (17.9)	<.001
De-escalated, N (%)	39 (12.4)	78 (22.7)	.002
De-escalation possible but not done, N (%)	18 (5.7)	21 (6.1)	
Length of hospital stay, days, median (25%–75%)	28.0 (16.0–46.0)	28.0 (15.0–47.5)	.678
Transfer to ICU during carbapenem therapy	39 (16.1)	52 (34.7)	<.001
Death			
In 7 days, N (%)	1 (0.03)	6 (1.7)	.125
In 30 days, N (%)	18 (5.5)	54 (15.3)	<.001
Colonization after carbapenem treatment, N (%)			
CR GNB	18 (45.0)	21 (33.9)	.259
*Candida* sp.	13 (32.5)	23 (37.1)	.635
*C. difficile*	2 (5.0)	2 (3.2)	.652
New episode of infection after carbapenem treatment, N (%)^c^	74 (27.6)	75 (28.3)	.859

a Analyzed for 286 patients hospitalized in the ICU.

b Includes both correct indication, dosage and duration of infusion.

c Analysis was performed on 102 patients who had records of at least 30-day follow-up in the hospital system after carbapenem was discontinued.

Note. Pre-GP: pre-guideline period; post-GP: post-guideline period; GNB; gram-negative bacilli; CR GNB; carbapenem-resistant gram-negative bacteria.

**Table 3.** Multivariate analysis of the effect of guideline implementation on appropriate carbapenem use, binary logistic regression

	OR	95% CI	*P* value
Post-guideline period	1.8	1.3–2.6	<.001
CCI	1.0	0.9–1.0	.685
COVID-19 history	5.0	2.1–12.1	<.001
ICU stay	1.8	1.3–2.6	.001
Blood culture growth	1.6	1.0–2.5	.051

Note. Hosmer and Lemeshow test *P* value = .814. IQR = interquartile range; OR = odds ratio; COVID-19 = coronavirus disease 2019.

Empirical carbapenem treatment was escalated in 37.4% of cases in the pre-GP compared to 17.9% in the post-GP (*P* = .004). Patients with diabetes mellitus (*P* = .021), hematological malignancies (*P* = .006), and those infected with SARS-CoV-2 (*P* < .001) required escalation more frequently. A higher proportion of escalated cases were in the ICUs compared to the wards (77.8% vs 52.9%; *P* = .001). Escalation was also higher in patients in whom source control could not be achieved due to unremoved infected catheters, undrained intra-abdominal or pulmonary abscesses/collections (*P* = .008). Forty-six percent of the patients in whom treatment was escalated were infected by carbapenem-resistant (CR) GNB.

There was no significant difference in the infection-related mortality between periods. However, 30-day mortality rate was significantly higher in post-GP (15.3%) compared to that in pre-GP (5.5%) (*P* < .001). Group comparisons are shown in Table 4. When CCI, COVID-19 history, length of ICU stay, blood culture growth, appropriate carbapenem use, new infection episode within 30 days were controlled by cox regression analysis, the guideline was not associated with mortality. CCI, COVID-19 history, ICU stay, new infection episode in 30 days were found to be associated with mortality (*P* values <.001, .005, .003, <.001, respectively) (Table 5).

**Table 4.** Evaluation of mortality-related factors in 30 days of carbapenem treatment

	Survived (N = 606)	Died (N = 72)	*P* value
Age (median-25%–75%)	64 (52–74)	65 (52–77)	.419
Women, N (%)	264 (43.6)	32 (44.4)	.887
Comorbidity, N (%)			
Diabetes mellitus	165 (27.2)	28 (38.9)	.038
Hypertension	231 (38.1)	35 (48.6)	.085
Cardiovascular disease	162 (26.7)	22 (30.6)	.490
Chronic renal disease	56 (9.2)	6 (8.3)	.801
Cirrhosis	6 (1.0)	3 (4.2)	.060
Rheumatological disease	20 (3.3)	2 (2.8)	1.000
Neurological disease	103 (17.0)	7 (9.7)	.113
Chronic pulmonary disease	80 (13.2)	7 (9.7)	.404
Solid tumor	218 (36.0)	33 (45.8)	.101
Hematological malignancy	76 (12.5)	8 (11.1)	.728
Hematopoietic stem cell transplantation (autologous and allogeneic)	17 (2.8)	–	
Solid organ transplantation	18 (3.0)	–	
COVID-19	54 (8.9)	20 (27.8)	<.001
Other	101 (16.7)	6 (8.3)	.067
Charlson comorbidity index (CCI), median (IQR; 25%–75%)	4 (2–7)	6.5 (4–8)	<.001^a^
Length of hospital stay, days, median (25%–75%)	28 (15–49)	27.5 (16.5–36.0)	.424
Ward where carbapenem was started, N (%)			
ICU	233 (38.4)	53 (73.6)	<.001
Non-ICU	373 (61.6)	19 (26.4)	
Transfer to ICU during treatment, N (%)	83 (22.3)	8 (42.1)	.054
Appropriate use of carbapenem, N (%)	218 (36.6)	30 (44.1)	.227
Modification of initial carbapenem treatment			
Escalated, N (%)	152 (25.1)	33 (45.8)	<.001
De-escalated, N (%)	109 (18.5)	8 (1.4)	.140
Positive blood culture, N (%)	100 (16.5)	8 (11.1)	.237
Carbapenem-resistant bacteria growth in cultures, N (%)	61 (10.1)	4 (5.6)	.219
Duration of carbapenem use, days, median (IQR, 25%–75%)	8 (5–12)	9 (6.0–11.5)	.638
Successful treatment of the infectious episode, N (%)	532 (87.8)	67 (93.1)	.188

Note. IQR: Interquartile range.

**Table 5.** Multivariate analysis of the effect of guideline implementation on 30-day mortality, cox regression

	HR	95% CI	*P* value
Post-guideline period	1.7	1.0–3.2	.066
CCI	1.1	1.1–1.2	<.001
COVID-19 history	2.3	1.3–4.2	.005
ICU stay	2.4	1.3–4.2	.003
Blood culture growth	0.8	0.4–1.7	.570
Appropriate use of carbapenem	0.8	0.4–1.3	.340
New infection episode in 30 days	3.3	2.1–5.4	<.001

Note. Omnibus test *P* value <.001. COVID-19: coronavirus disease 2019; HR: hazard ratio; CI: confidence interval.

Nosocomial bloodstream infection density rates with MDR bacteria were similar in both periods except for an increase in CRAB (Table 6). Consumption rates of carbapenems, piperacillin-tazobactam, colistin, tigecycline, and glycopeptides increased in the post-GP (Table 7). In contrast, consumption rate of sulbactam-ampicillin, cephalosporins, and fluoroquinolones were significantly lower for the same period compared with pre-GP.

**Table 6.** Comparison of infection density rates of nosocomial bloodstream infections caused by multidrug-resistant bacteria in pre-guideline and post-guideline periods

Bacteria	Infection density rate^a^ Pre-GP (%95 CI)	Infection density rate^a^ Post-GP (%95 CI)	Rate ratio (%95 CI)	*P* value
ESBL-EC	2.99 (2.34–3.77)	2.83 (2.11–3.72)	0.94 (0.65–1.36)	.842
ESBL-KP	1.23 (0.83–1.75)	1.00 (0.60–1.57)	0.81 (0.44–1.48)	.605
CRKP	3.30 (2.61–4.11)	4.26 (3.45–5.45)	1.32 (0.95–1.82)	.105
CRPA	1.89 (1.38–2.52)	1.77 (1.21–2.49)	0.93 (0.058–1.49)	.872
CRAB	2.11 (1.57–2.78)	4.07 (3.19–5.12)	1.92 (1.33–2.78)	<.001
MRSA	0.48 (0.25–0.84)	1.06 (0.64–1.64)	2.19 (1.03–4.64)	.056
VRE	0.39 (0.19–0.72)	0.29 (0.11–0.65)	0.74 (0.24–2.22)	.804

a Per 10,000 patient days.

Note. Pre-GP: pre-guideline period; post-GP: post-guideline period; CI: confidence interval; ESBL-EC: ESBL-producing *Escherichia coli*; ESBL-KP: ESBL-producing *Klebsiella pneumoniae*; CRKP: carbapenem-resistant *Klebsiella pneumoniae*; CRPA: carbapenem-resistant *Pseudomonas aeruginosa*; CRAB: carbapenem-resistant *Acinetobacter baumannii*; VRE: vancomycin-resistant *Enterococcus faecium*.

**Table 7.** Consumption antibiotic consumption rated per 1,000 patient days in pre-guideline and post-guideline periods

	Consumption rate			
Antibiotics	2018/Pre-GP (%95 CI)	2020/Post-GP (%95 CI)	Rate ratio (%95 CI)	*P* value
Meropenem and imipenem	18.75 (18.2–19.31)	28.91 (28.22–29.61)	1.542 (1.484–1.602)	<.001
Ertapenem	3.799 (3.55–4.06)	3.51 (3.28–3.76)	0.926 (0.841–1.019)	.114
Piperacillin-tazobactam	13.46 (13–13.94)	14.85 (14.36–15.36)	1.103 (1.051–1.158)	<.001
Ampicillin-sulbactam	27.95 (27.28–28.64)	18.4 (17.86–18.97)	0.658 (0.633–0.684)	<.001
3^rd^ and 4^th^ generation cephalosporins	10.13 (9.73–10.55)	9.27 (8.88–9.67)	0.91 (0.86–0.97)	.003
Fluoroquinolones	13.57 (13.1–14.05)	5.98 (5.67–6.30)	0.44 (0.41–0.46)	<.001
Amikacin	21.06 (15.75–27.61)	26.56 (20.49–33.89)	1.26 (0.86–1.83)	.263
Colistin	5.72 (5.41–6.03)	8.16 (7.80–8.54)	1.42 (1.33–1.53)	<.001
Tigecycline	3.53 (3.29–3.78)	8.07 (7.71–8.45)	0.45 (0.42–0.49)	<.001
Vancomycin and teicoplanin	11.02 (10.6–11.4)	14.8 (14.3–15.3)	1.35 (1.28–1.42)	<.001
Linezolid and daptomycin	0.99 (0.87–1.12)	1.36 (1.22–1.52)	1.37 (1.16–1.63)	<.001

Note. Third-and fourth-generation cephalosporins: cefotaxime, ceftriaxone, ceftazidime, cefepime; fluoroquinolones: ciprofloxacin, levofloxacin, moxifloxacin. Pre-GP: pre-guideline period; post-GP: post-guideline period; CI: confidence interval.

## Discussion

In this study, we found that the implementation of hospital-specific guidelines alone had a positive effect on the appropriate use of carbapenems. This was also seen in patients with bacteraemia. The improvement in the use of carbapenems was not only limited to the choice of the appropriate indication, but also to the dosage strategies. There was a 2.3-fold increase in the use of prolonged infusion and correct initial dose of carbapenems in ICU patients with renal impairment after the hospital guidelines were introduced. De-escalation has been encouraged as important component of antimicrobial stewardship protocols and a statistically significant increase in the rate of de-escalation was observed after the introduction of the guidelines (Table 2). This is remarkable finding at an institution with a high endemicity for MDR bacteria.

Local guidelines for diagnosis, treatment, and follow-up of infectious conditions are essential for standardized patient management in accordance with current medical principles. This is especially important for both correct clinical approach of the junior residents during night calls and for training purposes. Construction of a local guideline should encompass evidence-based data from the literature and recommendations of widely accepted international societies or organizations. In addition, blending the existing data with local resistance profiles as well as available local resources is imperative. A local guideline also provides the basis for assessment of the quality of care provided for that specific condition, most frequently as audit and feedback.

Although carbapenem use was more appropriate after the local guidelines, this did not result in an increased success rate in the treatment of the infectious episode (89.0% vs 92.0%, *P* = .169). This lack of effect could be attributed to several factors, mainly the presence of unfavorable conditions in the post-GP such as the type of infection (urinary tract infection vs pneumonia), more patients in the ICU, and higher CCI values.

Carbapenem treatment was escalated more frequently in pre-GP compared to post-GP. This reflects the appropriateness of pre-defined criteria for carbapenem use in the guidelines. Yet, 17.9% escalation rate suggests some improvement may still be achieved in terms of better coverage in the initial treatment regimen. The variables associated with escalation were significant for COVID-19, ICU admission, and uncontrolled infection source. It has been shown that the SARS-CoV-2 pandemic had a negative effect on health care; more infections caused by MDR bacteria and more frequent use of antibiotics were observed. We reported similar findings in a recent study where we examined the impact of COVID-19 pandemic on bloodstream infections caused by MDR bacteria and antibiotic consumption between 2018 and 2020.^12^ Infection density rates of MDR bacteria were the lowest at the start of the pandemic (the first half of 2020), but then increased rapidly which triggered the increased rate of meropenem consumption, especially in the COVID-ICUs. Active surveillance was suspended between March 2020 and April 2021 due to the COVID-19 pandemic, an outbreak of MDR *A. baumannii* was detected in COVID ICUs through a laboratory-based analysis of bloodstream infection surveillance in November 2020, which may also contribute to escalation of carbapenem treatment.

Most carbapenem stewardship studies have reported no change in mortality despite an improvement in antimicrobial use.^13–15^ After adjustment for CCI, COVID-19 history, ICU stay and blood culture growth, appropriate use of carbapenems and new infection episode in 30 days, the guideline did not have an influence on mortality in our study, either. However, Spernovasilis et al. found a reduction in mortality when a carbapenem-focused antimicrobial stewardship program was implemented during the COVID-19 pandemic in a setting of high endemicity for MDR GNB.^16^ In contrast to this study, in our study antibiotic use was restricted with the approval of the ID physician, including the pre-GP period. Also, unlike the other study, the effect of the guideline alone was assessed without audit feedback. The difference between the studies may be due to differences in both the intervention and control groups.

Our study is unique in that it evaluates the effect of local guidelines with no further interventions on carbapenem use. Most early studies have shown a favorable influence of a guideline on patient care, such as in the treatment of community-acquired^17–20^ or health-care-associated pneumonia,^21–23^ febrile neutropenia,^24^ skin and soft tissue infections,^25^ and staphylococcal bacteremia^26^ with no specific focus on carbapenems. More recent studies have utilized various interventional approaches for optimal carbapenem use.^13–16^ Garcia-Rodriguez et al reported their experience with the introduction of local guidelines, auditing, and feedback.^3^ Their approach resulted in an increase in the eligibility of carbapenem prescription (from 49.7% in 2015 to 80.9% in 2019), and a subsequent decrease in carbapenem consumption while consumption of cefepime and piperacillin increased. This was accompanied by a decrease in the overall frequency of bacteremia caused by MDR bacteria. This latter finding is in contrast to the result of our study. It should be emphasized that the prevalence of MDR bacteria in a particular healthcare facility depends on several factors other than the availability of antimicrobial stewardship protocols and appropriate use of antimicrobials, such as effective infection prevention and control measures, availability of narrow-spectrum antibiotics in the hospital pharmacy, timely transfer of patients from the ICU to the ward, effective transfer of patients requiring palliative care to nursing homes and end-of-life care facilities, and finally the burden of a pandemic on healthcare workers.

Our study has several limitations. First, retrospective data collection relies on the hospital’s electronic system, which may introduce potential bias. Second, routine documentation of some confounding factors, such as infection control practices and information on clustering and mini-epidemics in the hospital may not optimal, especially during the COVID-19 pandemic. Due to the lack of randomization, the effect of unmeasured confounders cannot be completely excluded. As the level of hospital-wide compliance with the guideline is not known, the impact of the guideline may have been underestimated.

In conclusion, introduction of hospital-specific guidelines alone improves appropriate use of carbapenems even when antimicrobial treatment is mainly regulated by ID Department. A multifaceted carbapenem-focused ASP together with an effective infection control program may result in desired reductions in all-cause mortality rates, and prevalence of MDR pathogens.

## Supporting information

Boşnak et al. supplementary material Boşnak et al. supplementary material

## Supplementary material

For supplementary material accompanying this paper visit http://dx.doi.org/10.1017/ash.2024.415. click here to view supplementary material

## Research transparency and reproducibility

The data that support the findings of this study are available from the corresponding author upon reasonable request. Due to privacy and ethical considerations, access to the data will be granted on a case-by-case basis, ensuring that the request is aligned with the research objectives and ethical standards.
