# Supplementary figures and images for: The impact of hospital-specific guidelines on carbapenem use and patient outcomes in a setting for high endemicity with multidrug-resistant gram-negative bacilli
Source: Antimicrob Steward Healthc Epidemiol. 2024 Sep 25;4(1):e146. doi: 10.1017/ash.2024.415 (PMC11428004; doi:10.1017/ash.2024.415)

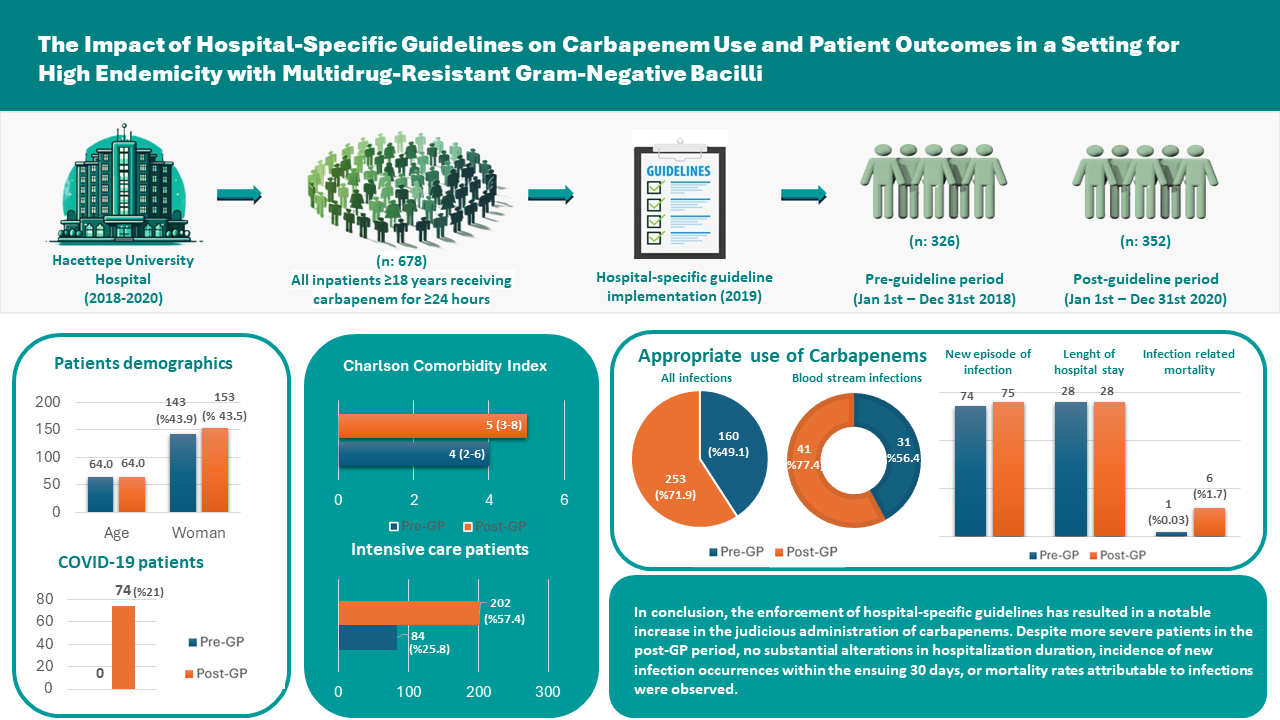

Supplement: Boşnak et al. supplementary material [file S2732494X24004157sup001.tif]
